# Heterocycles in Peptidomimetics and Pseudopeptides: Design and Synthesis ^†^

**DOI:** 10.3390/ph5030297

**Published:** 2012-03-08

**Authors:** Iole Cerminara, Lucia Chiummiento, Maria Funicello, Ambra Guarnaccio, Paolo Lupattelli

**Affiliations:** Dipartimento di Chimica “Antonio Mario Tamburro”, Università degli Studi della Basilicata, Via Ateneo Lucano 10, Potenza 85100, Italy; Email: iole.cerminara@gmail.com (I.C.); lucia.chiummiento@unibas.it (L.C.); ambra.guarnaccio@gmail.com (A.G.); paolo.lupattelli@unibas.it (P.L.)

**Keywords:** heterocycles, peptidomimetics, tricyclic compounds, scaffold

## Abstract

This minireview provides a brief outline of the peculiar aspects of the preparation of peptidomimetic and pseudopeptidic structures containing heterocycles. In particular novel tricyclic structures are investigated as potential drugs.

## 1. Introduction

Heteroarenes are widely known in many fields of organic chemistry; in particular medicinal chemistry is intimately associated with heterocyclic compounds and most known chemicals used in medicine are based on heterocyclic frameworks [1].

There are two main sources of heteroarenes: They abound in Nature, often in complex form, but they can also be prepared in research laboratories by different synthetic approaches. Herein we generally describe synthesis and elaboration of benzocondensed heteroarenes and their introduction in peptidomimetic structures. In particular, we focus on benzothiophenes with a view to preparation of benzothienopyridines and their potential application as scaffolds in pseudopeptides.

## 2. Peptidomimetic Structures

Peptidomimetics typically are small protein-like molecules designed to mimic natural peptides or proteins. These mimetics, whose structures were mainly derived from natural peptides, should have the ability to bind to their natural targets in the same way of the natural sequences and hence should produce the same biological effects.

It is possible to design these molecules in such a way that they show the same biological effects as their peptide role models, but with enhanced properties like a higher proteolytic stability, higher bioavailability and also often with improve selectivity or potency [2].

Moreover it is known that the isosteric replacement of a peptide bond represents an important and general tool in design of peptidomimetics together with the incorporation of conformationally restricted units, such as rings, into a peptide sequence to force it to adopt a known, biologically active conformation [3].

The goal is to replace as much of the peptide backbone as possible with non-peptide fragments while still maintaining the pharmacophoric groups (usually the amino acid side chains) of the peptide. This makes the compound more lipophilic, and therefore its bioavailability is increased. Replacement of the amide bond with alternative groups prevents proteolysis and promotes metabolic stability. Initially, conformational flexibility has to be retained to allow the pharmacophoric groups a better opportunity to find their binding sites, but further lead refinement should favour the formation of more conformationally restricted analogs that hold appropriate pharmacophoric groups in the bioactive conformation for binding to the target receptor [4].

There are many examples in literature of peptidomimetics that result from the incorporation of a heterocycle into a peptide, or peptide-like structure; the pre-organization of peptide shape, via the introduction of a structural motif that imparts conformational restriction, can enhance binding and hence therapeutic potential [5].

**Figure 1:**
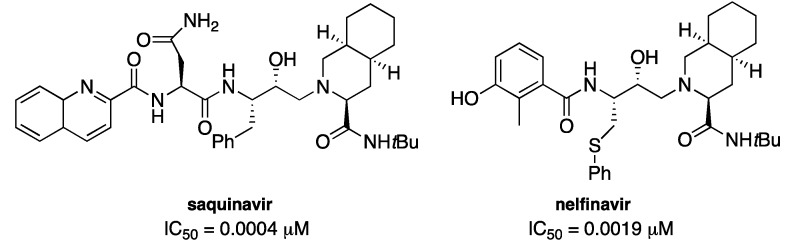
Structures of saquinavir and nelfinavir.

To the best of our knowledge many drugs are peptidomimetics, in particular drugs actually in use for the inhibition of HIV protease (HIV-Pr) [6]. To date [7] there are ten commercially available drugs that target the HIV-Pr and nine of these collectively represent a success story in rational molecular design of peptidomimetics. Among these we focused our attention on saquinavir and nelfinavir, attracted by the great activity of the first and from the presence of a sulfur atom in the core of the second (Figure 1).

Our idea was the possibility of change in the core of this class of peptidomimetics by replacing of the phenyl ring with a heterocyclic ring, namely thiophene or benzothiophene: In fact it is well known that a thienyl ring mimics a phenyl group of phenylalanine in peptidomimetics [8] and in many drugs [9,10,11].

**Figure 2:**
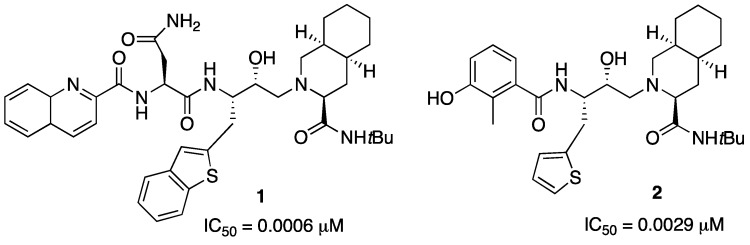
Structures of thienyl and benzothienyl derivatives of saquinavir (**1**) and nelfinavir (**2**).

On the basis of our experience in the chemistry of thiophene and benzothiophene [12,13] as well as in stereoselective synthesis [14,15], we began to work toward the synthesis of new heterocycle containing structures as potential inhibitors of HIV-Pr [16]. From this research, after different synthetic approaches, we were able to prepare the reported structures (Figure 2): It was remarkable that such compounds were active either against the wild type of protease or the mutated one.

**Scheme 1:**
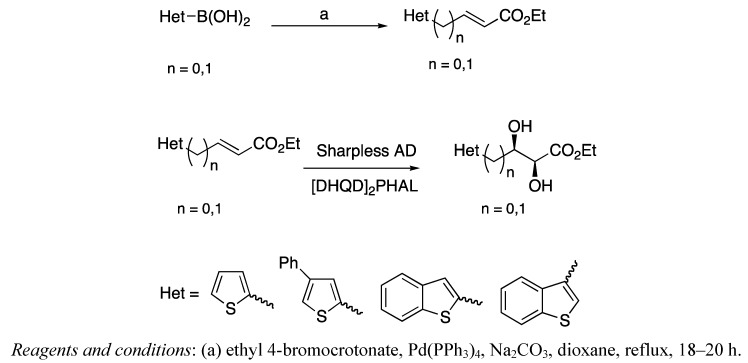
Suzuki coupling and AD reaction.

The goal in this synthetic approach was the construction of the core starting from two important and actual reactions: A Suzuki coupling for the introduction of the heterocyclic ring and the Sharpless asymmetric dihydroxylation (AD) for the introduction of the correct chirality in the core of the potential inhibitor [17,18] (Scheme 1).

**Figure 3:**
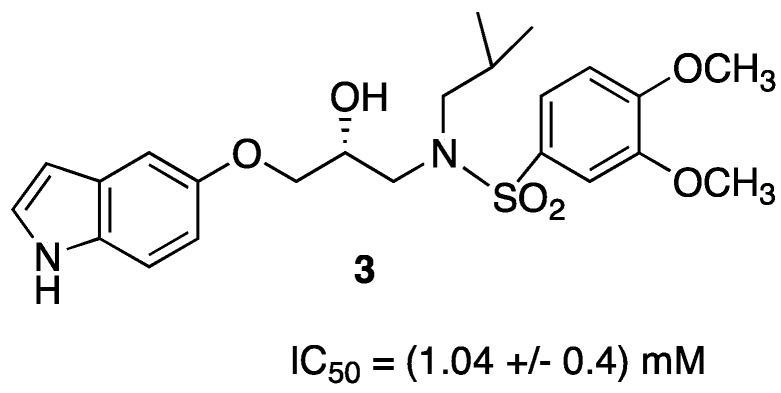
Structure of indolic non-peptidic HIV protease inhibitor.

Furthermore, the need to overcome viral resistance and to have a simpler synthetic sequence prompted us to investigate new non-peptidic compounds, with simplified structure, bearing the central isopropanolamine unit found in the commercially available peptidic drugs [19,20,21]. In our continuing interest on new inhibitors containing heteroarylic groups, the attention focused on some reported structures [22] in which the indole ring, mimicking the aminoindane group of Indinavir, seems to be important for the activity. So we produced another series of inhibitors [23] containing an indole ring in P2 position: Unfortunately the activity of these compounds remained in the range of micromolar scale, even if SAR studies showed the importance of the indolic NH presence in that position for a good interaction with active site of the protease (Figure 3).

## 3. Pseudopeptides

Another approach to construct peptidomimetics involves the design of conformationally restricted analogs that mimic and/or stabilise characteristics of the receptor-bound conformation of the endogenous peptide, such as β-turns and other.

The ability to access to such structures modified by incorporation of heterocycles allows the study of the associated biological processes, with an opportunity for further drug design and development. 1,2,3-Triazoles, for example, offer an appealing structural motif in peptidomimetic research because their structural and electronic characteristics are similar to those of a peptide bond [24]. It is also known that secondary structures (β-turns, α-helices, β-strands) are sites of recognition by the enzymes, such as proteases [25].

This idea can be extended to *scaffold peptidomimetics* in which important pharmacophoric residues are held in the appropriate orientation by a rigid template. So in this field, much effort has been devoted to the design and synthesis of conformationally constrained compounds that mimic, or induce, specific secondary structural features of peptides and proteins.

Many scaffolds possessing the functionalities of peptides (*i.e*., amine and carboxyl groups) together with well defined spatial properties, thus reproducing the desired orientation of a growing peptide chain, have hence been created [26].

**Figure 4:**
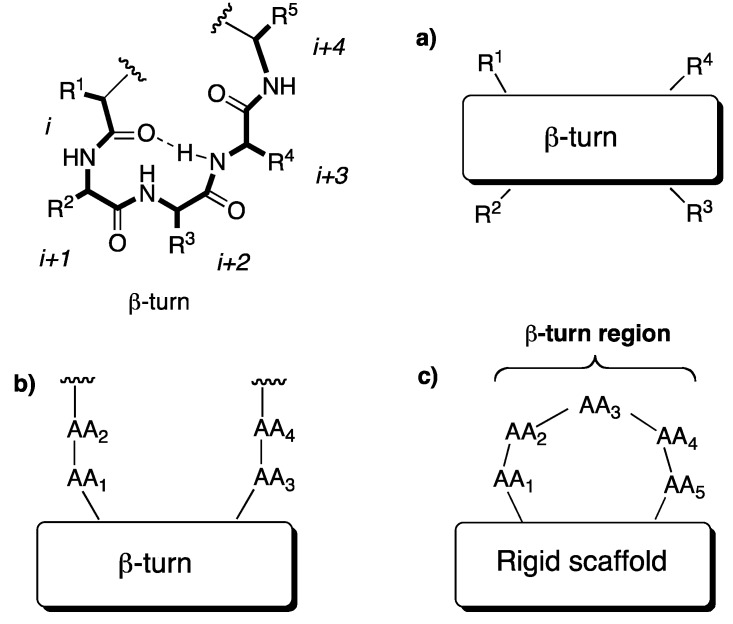
β-turn and induced β-turn. (**a**) internal β-turn mimics; (**b**) β-hairpin mimics (in which a rigid scaffold induces reversal of the peptide chain); (**c**) external β-turn inducers.

The β-turn is a common feature in biologically active peptides and is defined as any tetrapeptide sequence, with a 10-membered intramolecularly H-bonded ring, in which the C_α_(i) to C_α_(i+3) distance varies from 4 to 7 Angstrom (Figure 4).

There are at least 14 types of β-turn structures, described in literature [26]. These conformers used as models have been developed for linear and short peptides. In natural proteins, turn fragments can adopt an even larger variety of conformations, due to stabilization provided by the remaining portion of molecule. Although there has been much discussion in the literature on what constitutes a β-turn mimic and how different types of mimics have to be characterized, these can be roughly classified into three broad classes, illustrated in Figure 4.

Aiming to construct a β-turn mimic with a heterocyclic scaffold we hypothesized an opportune synthetic approach, in particular directed to the preparation of potential active structure for the treatment of Alzheimer’s disease, either as β-secretase or as β-amyloid aggregation inhibitor.

β-Secretase (memapsin 2 or BACE1), discovered in 1999 [27,28], is one of the two proteases that cleave the β-amyloid precursor protein (APP) to produce a 40–42 residue amyloid β-peptide (Aβ) in the human brain. Accumulation of Aβ results in the formation of amyloid plaques and neurofibrillary tangles. The neurotoxicity of Aβ is ultimately responsible for brain inflammation, neuronal death, dementia and Alzheimer’s disease [29]. Consequently therapeutic inhibition of memapsin 2 has emerged as one of the most active areas of today’s drug development for the intervention of Alzheimer’s disease.

**Figure 5:**
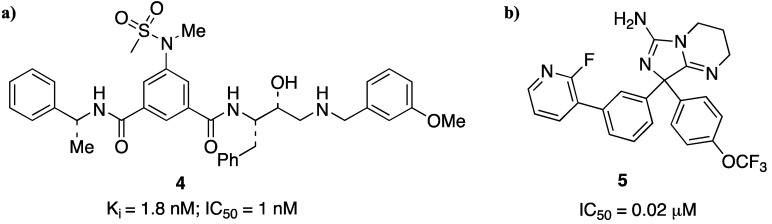
Two potential β-secretase inhibitors. (**a**) GRL-8234; (**b**) heteroarylaminoimidazole.

It is known that β-secretase is an aspartic protease, for which the inhibition mechanism and the design of transition state analogues through the successful development of HIV-1 protease inhibitor drugs are well previously described [30]. So, as first approach to the design and synthesis of potential inhibitors, Ghosh and co-authors reported [31] a series of Leu-Ala isosteres which led to the first potent transition-state inhibitor (CTS-21166) that is actually in advanced clinical trials. This compound (an optimization of the structure is reported in Figure 5a) represents the first small peptidomimetic inhibitor modifying therapy for Alzheimer’s disease.

The newest generation BACE1 inhibitors are low molecular weight molecules with excellent cell permeability, have little or no peptidic character, and possess enhanced pharmacokinetic profiles [32,33,34,35]. Actually many lines of research are concerned with the synthesis and study of small-molecule-containing heterocyclic rings as β-secretase inhibitors [36,37] (Figure 5b).

## 4. Scaffold for Pseudopeptides: Design and Synthesis of Heteroaromatic Tricyclic Structures

**Figure 6:**
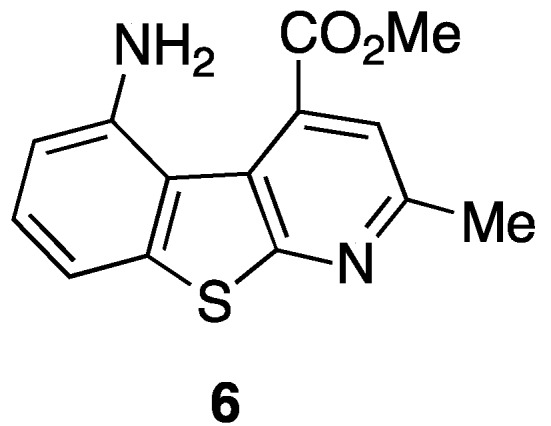
5-Amino-4-carbomethoxy-2-methylbenzothienopyridine.

Our long interest in the preparation of heterocycles and their application in medicinal chemistry prompted us to design and synthesize new tricyclic compounds as potential scaffolds for pseudopeptides and/or as β-secretase inhibitors. Recently [38], a particular structure that mimics a biological turn has just been synthetized in our laboratory. The molecule is shown in Figure 6.

**Scheme 2:**
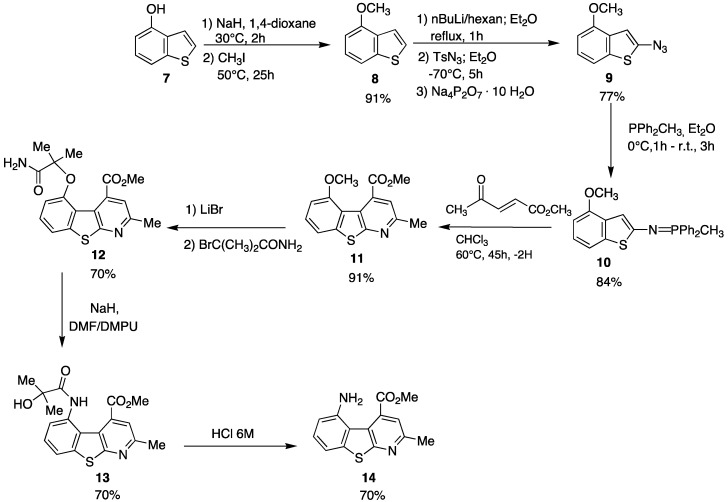
Synthetic route affording 5-amino-4-carbomethoxy-2-methylbenzothienopyridine.

As can be seen such a benzothienopyridine bears two interesting groups in the 4- and 5-positions of the tricyclic structure, a carbomethoxy and an amine function, respectively, that immediately suggest its possible use as a pseudopeptide scaffold as well as a β-secretase inhibitor. For its preparation an innovative synthetic route was developed (Scheme 2).

The depicted synthetic approach is based on four principal reactions:

(1) An azido-transfer reaction that furnishes the azido precursors [39];
(2) A Staudinger reaction that transforms the azido group into an iminophosphorane, a powerful tool for the construction of nitrogen containing heterocycles [40,41,42,43,44]
(3) A tandem aza-Wittig/electrocyclization of iminophosphoranes with suitable α,β-unsaturated carbonyl compounds;
(4) Finally, a Smiles rearrangement that affords the benzothienopyridine [45,46,47,48]

It is well known that benzothienopyridines are of pharmacological interest due to their isosterism with indolopyridines [49,50] and to their reported activity as antibacterial [51], antiallergic [52] and anxiolytic agents [53], so the development of a facile preparation seems to be of timely interest.

**Scheme 3:**
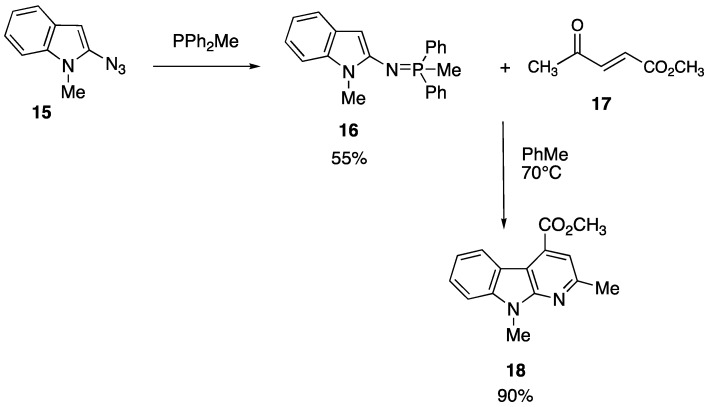
Synthesis of α-carboline.

Our experience in heterocyclic chemistry suggested us also to extend the use of Staudinger reaction (and subsequent aza-Wittig/electrocyclization reaction) either to indole, benzofuran or to substituted benzothienyl ring. As an example of such a methodology for the preparation of a benzofuropyridine [54] and α-carboline [55] we reported the reaction scheme realized for 2-azido-*N*-methylindole (Scheme 3).

**Scheme 4:**
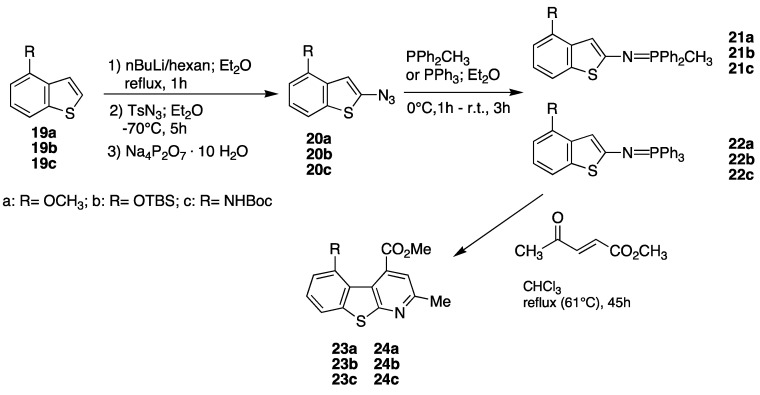
Synthesis of 5-amino (or hydroxy)-4-carbomethoxy-2-methylbenzothienopyridines.

**Scheme 5:**
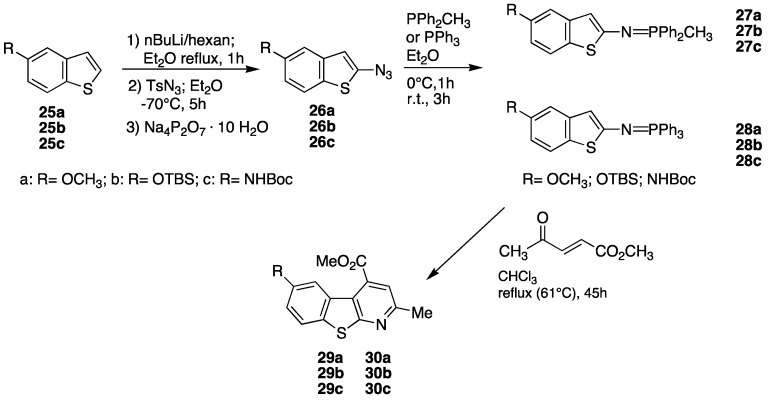
Synthesis of 4-carbomethoxy-6-hydroxy-(or amine)-2-methylbenzothienopyridines.

This efficient strategy was then applied on 4- and 5-substituted benzothiophenes; preliminary results are shown in the next Scheme 4 and Scheme 5, and Table 1 and Table 2, [56,57].

**Table 1:** Synthesis of 5-amino (or hydroxy)-4-carbomethoxy-2-methylbenzothienopyridines.

Entry	Azide (%yield)	Phosphorane (%yield)	Benzothienopyridine (%yield)
**1**	20a (77%)	21a (84%)	23a (91%)
**2**	20b (80%)	21b (40%)	23b (14%)
**3**	20c (30%)	21c (no product)	23c (no product)
**4**	20a (77%)	22a (33%)	24a (40%)
**5**	20b (80%)	22b (20%)	24b (no product)
**6**	20c (30%)	22c (no product)	24c (no product)

**Table 2:** Synthesis of 4-carbomethoxy-6-hydroxy-(or amine)-2-methylbenzothienopyridines.

Entry	Azide (%yield)	Phosphorane (%yield)	Benzothienopyridine (%yield)
**1**	26a (55%)	27a (58%)	29a (35%)
**2**	26b (28%)	27b (79%)	29b (13%)
**3**	26c (62%)	27c (49%)	29c (no product)
**4**	26a (55%)	28a (74%)	30a (52%)
**5**	26b (28%)	28b (81%)	30b (58%)
**6**	26c (62%)	28c (no product)	30c (no product)

As can be seen, the presence of a *t-*butyldimethylsilylether functionality in the 4-position of the benzothiophene plays an important different effect: In fact the azido transfer reaction was favoured, while the Staudinger reaction was unfavoured, in particular when triphenylphosphine was used.

Probably, such an effect was due to the hindrance of the substituent either on phosphorous or on oxygen. In presence of a methoxy group at the 4-position of benzothiophene the effect on the azido formation was the same (we hypothesized a predominant electronic effect), but it had the opposite effect on iminophosporane synthesis.

The NH-Boc substituent merits particular: In this case the presence of the hydrogen on the *N*-function represents a drawback during lithiation, despite the hindrance of the *t*-butyl group; hence the chemical yields were unsatisfactory, even with excess of butyl lithium.

A different result was obtained when the same synthetic route was used on 5-substituted benzothiophene, precursor of 4-carbomethoxy-6-hydroxy-(or amine)-2-methylbenzothienopyridine (Figure 11). As can be observed it seems that electronic effects were prevalent for all considered substrates, both in the azido transfer and in the Staudinger reaction leading to iminophosphoranes, but the final cyclization is still not an optimized procedure. Further studies on the synthesis of the described tricyclic structures and their potential biological activity on β-secretase are in progress.

## 5. Experimental

### 5.1. General

Column chromatography was carried out on Merck silica gel (0.063–0.200 mm particle size) by progressive elution with petroleum ether/ethyl acetate or petroleum ether/diethyl ether mixtures. ^1^H- and ^13^C-NMR spectra were normally recorded for CDCl_3_ solutions on a Bruker AM 300 MHz or on Varian INOVA 400 and 500 MHz instruments. IR spectra were registered on a JASCO FT/IR 460 Plus. Mass spectra were obtained with a Hewlett-Packard 5971 mass-selective detector on a Hewlett-Packard GC/MS 6890-5973 system. Dichloromethane, chloroform and carbon tetrachloride were dried with anhydrous CaCl_2_; diethyl ether and 1,4-dioxane were dried using sodium/benzophenone. Dry dimethylformamide was commercially available. 4-Hydroxybenzo[*b*]thiophene was prepared according to the literature [38]. 2-Bromo-2-methylpropanamide was synthesized as recently reported [38] and used in Smiles rearrangement as reported in the same paper to obtain the suitable 2-aryloxy-2-methylpropanamide. 5-Aminobenzo[*b*]thiophene was commercially available (Maybridge Trevillet, Tintagel, Cornwall, UK).

### 5.2. Synthesis of 4,5-Disubstituted Benzothieno[2,3-b]pyridine Precursors

*4-Methoxybenzo[b]thiophene* (**19a**). Compound **19a** was synthesized from 4-hydroxybenzo[*b*]-thiophene according to a known procedure [38]. Thick red oil (yield 91%). Found: C, 65.85; H, 4.88; S, 19.56%. C_9_H_8_OS requires C, 65.83; H, 4.91; S, 19.52%; ^1^H-NMR (300 MHz, CDCl_3_) (ppm): δ_H_ 7.65–7.5 (m, 2H), 7.45–7.38 (m, 2H), 6.84–6.80 (m, 1H), 3.99 (s, 3H); ^13^C-NMR (75 MHz, CDCl_3_) (ppm): δ_C_ 155.0, 141.2, 130.5, 125.3, 124.6, 120.5, 115.0, 104.1, 55.9; MS *m/z*: 164 (M^+^), 149 (100).

*4-(Tert-butyldimethylsilyloxy)benzo[b]thiophene* (**19b**). Compound **19b** was synthesized from 4-hydroxybenzothiophene according to a known procedure [38]. Thick oil (yield 75%). Found: C, 63.59; H, 7.59; S, 12.15%. C_14_H_20_OSSi requires: C, 63.58; H, 7.62; S, 12.12%; ^1^H-NMR (300 MHz, CDCl_3_) (ppm): δ_H_ 7.72–7.67 (m, 2H), 7.53–7.37 (m, 2H), 6.99–6.93 (m, 1H), 1.35 (s, 9H), 0.50 (s, 6H); ^13^C-NMR (75 MHz, CDCl_3_) (ppm): δ_C_ 150.7, 141.5, 133.5, 125.3, 124.3, 121.0, 115.7, 113.1, 25.7, 18.5, 20.4; MS *m/z*: 264 (M^+^), 207 (100).

*Tert-butyl benzo[b]thiophen-4-yl carbamate* (**19c**). 4-Hydroxybenzo[*b*]thiophene (135 mg, 0.9 mmol) was converted to *N*-(benzo[*b*]thiophen-4-yl)-2-hydroxy-2-methylpropanamide (190 mg, 90%) via a Smiles rearrangement according to a known procedure [38]. The *N*-(benzo[*b*]thiophen-4-yl)-2-hydroxy-2-methylpropanamide was dissolved in HCl 6M (10 mL) and the resulting mixture was stirred at 100 °C for 4 h. After cooling to 25 °C the mixture was slowly treated with a solution of NaOH 2M until neutrality, then extracted twice with diethyl ether and dried over sodium sulphate. Removal of the solvent gave the 4-aminebenzo[*b*]thiophene as a black thick oil (107 mg, 90%).

To a solution of 4-aminobenzo[*b*]thiophene (107 mg, 0.72 mmol) in dry dichloromethane (4 mL), di-*tert*-butyl dicarbonate (BOC) (205 mg, 0.94 mmol) was added and the resulting mixture was stirred in an inert atmosphere for 15 h at room temperature [56]. After solvent removal, the crude was dissolved into ethyl acetate and the organic phase was washed several times with water, once with brine and then dried over sodium sulphate and finally concentrated under vacuum. The crude product was chromatographed on silica gel, using petroleum ether/ethyl acetate 7:3 as eluent, to give **19c** as a thick light pink oil (108 mg, 60%). ^1^H-NMR (400 MHz, CDCl_3_) (ppm):δ_H_ 7.82–7.80 (m, 1H), 7.60–7.58 (m, 1H), 7.43–7.41 (m, 1H), 7.33–7.30 (m, 1H), 6.72 (s, 1H), 1.54 (s, 9H); ^13^C-NMR (125 MHz, CDCl_3_) (ppm): δ_C_ 152.9, 140.5, 132.7, 131.1, 125.9, 124.9, 119.1, 117.8, 115.1, 80.8, 28.3; MS: *m/z* 249 (M^+^); 193 (100).

### 5.3. Synthesis of Azides ***20a,b,c***

*2-Azido-4-methoxy-1-benzo[b]thiophene* (**20a**). Compound **20a** was synthesized from **19a** according to a known procedure [38]. Thick yellow oil (yield 77%). Found: C, 52.65; H, 3.47; N, 20.45; S, 15.63%. C_9_H_7_N_3_OS requires: C, 52.67; H, 3.44; N, 20.47; S, 15.62%; IR ν_max_/cm^−1^ 2113 (N_3_); ^1^H-NMR (300 MHz, CDCl_3_) (ppm): δ_H_ 7.35-7.20 (m, 2H); 7.05 (s, 1H); 6.80-6.75 (m, 1H); 3.95 (s, 3H); ^13^C-NMR (75 MHz, CDCl_3_) (ppm): δ_C_ 152.7, 139.5, 138.2, 125.3, 124.6, 117.1, 115.7, 110.3, 50.5.

*[(2-Azidobenzo[b]thiophen-4-yl)oxy](tert-butyl)dimethylsilane* (**20b**). Compound **20b** was synthesized from **19b** according to a known procedure [38]. Thick yellow oil (yield 80%). Found: C, 55.07; H, 6.21; N, 13.78; S, 10.44%. C_14_H_19_N_3_OSSi requires C, 55.05; H, 6.27; N, 13.76; S, 10.50%; IR ν_max_/cm^−1^ 2110 (N_3_); ^1^H-NMR (300 MHz, CDCl_3_) (ppm): δ_H_ 7.50–7.40 (m, 1H), 7.19–7.11 (m, 1H), 6.89 (s, 1H), 6.72–6.68 (m, 1H), 1.07 (s, 9H), 0.27 (s, 6H); ^13^C-NMR (75 MHz, CDCl_3_) (ppm): δ_C_ 142.7, 125.3, 124.5, 121.0, 115.6, 115.0, 113.6, 108.3, 25.8, 15.9.

*Tert-butyl (2-azidobenzo[b]thiophen-4-yl)carbamate* **20c**. Compound **19c** (108 mg, 0,43 mmol) in dry diethyl ether (7 mL) was treated, under an inert atmosphere, with 1.6 M *n*-butyl lithium in hexane (0.65 mmol) and refluxed for 1 h. Then the reaction mixture was cooled to −70 °C and, slowly, tosylazide (119 mg, 0.60 mmol) in dry diethyl ether (3 mL) was added. After 5 h at this temperature the obtained triazene salt was filtered under vacuum, washed with dry diethyl ether and treated at 0 °C with an aqueous solution of sodium pyrophosphate decahydrate (316 mg, 0.7 mmol, in 5 mL of water). After 15 min of stirring at this temperature the suspension was filtered on a Buckner funnel and extracted twice with diethyl ether and then with ethyl acetate. After solvent removal, the crude product was purified by chromatography on Florisil (eluent petroleum ether/ethyl acetate 8:2) giving the title compound (38 mg, 30%) as a thick light yellow oil. Compound **19c** is unstable, so it was only characterized by IR and ^1^H-NMR spectroscopy. IR ν_max_/cm^−1^ 2125 (N_3_); ^1^H-NMR (400 MHz, CDCl_3_) (ppm): δ_H_ 7.73 (s, 1H), 7.55–7.53 (m, 1H), 7.38–7.36 (m, 1H), 7.27–7.24 (m, 1H), 6.66 (s, 1H), 1.49 (s, 9H).

### 5.4. Synthesis of Imminophosphoranes ***21a,b,c-22a,b,c***

*[(4-Methoxy-1-benzothiophen-2-yl)imino]-(methyl)diphenylphosphorane* **21a**. This compound was prepared from the azide **20a** and methyldiphenylphosphine (1eq) according to a known procedure [38]. Chromatography with petroleum ether/ethyl acetate 7:3 as eluent gave the title compound (yield 84%) as a red oil. Found: C, 70.04; H, 5.36; N, 3.69; S, 8.50%. C_22_H_20_NOPS requires C, 70.01; H, 5.34; N, 3.71; S, 8.49%; ^1^H-NMR (300 MHz, CDCl_3_) (ppm): δ_H_ 7.62–7.50 (m, 5H), 7.43–7.32 (m, 5H), 6.98–6.90 (m, 1H), 6.87–6.80 (m, 1H), 6.53–6.48 (m, 1H), 6.08 (s, 1H), 3.70 (s, 3H), 2.25–2.20 (d, 3H, *J_PH_* = 12.8 Hz); ^13^C-NMR (75 MHz, CDCl_3_) (ppm): δ_C_ 157.0, 152.3, 140.2, 135.3, 133.0, 132.5, 132.0, 131.7, 131.4, 130.0 129.5, 128.2, 125.8, 121.0, 114.3, 101.5, 55.5, 15.5.

*4-(Tert-butyldimethylsilyl)oxy)-N-(methyldiphenylphosphoranyliene)benzo[b]thiophen-2-amine* **21b**. This compound was prepared from the azide **20b** and methyldiphenylphosphine (1eq) according to a known procedure [38]. Chromatography with petroleum ether/ethyl acetate 7:3 as eluent gave the title compound (88 mg, 40%) as a brown powder, mp 90–92 °C (diethyl ether). (Found: C, 67.66; H, 6.79; N, 2.99; S, 6.73%. C_27_H_32_NOPSSi requires C, 67.89; H, 6.75; N, 2.93; S, 6.71%); ^1^H-NMR (300 MHz, CDCl_3_) (ppm): δ_H_ 7.83–7.77 (m, 5H), 7.58–7.37 (m, 5H), 7.11–7.06 (m, 1H), 6.87–6.77 (m, 1H), 6.52–6.49 (m, 1H), 6.02 (s, 1H), 2.25–2.20 (d, 3H, *J_PH_* = 12.8 Hz); 0.95 (s, 9H), 0.1 (s, 6H); ^13^C-NMR (75 MHz, CDCl_3_) (ppm): δ_C_ 168.3, 151.7, 147.5, 135.8, 134.7, 134.0, 132.2, 131.2, 130.8, 129.5, 129.0, 128.8, 120.9, 116.1, 113.2, 101.9, 25.7, 18.3, 14.5, 13.8.

*Tert-butyl 2-(methyldiphenylphosphoranylidene)aminobenzo[b]thiophen-4-yl)carbamate* **21c**. Compound **20c** (62 mg, 0.21 mmol) in dry diethyl ether (3 mL) was added dropwise to a solution of methyldiphenylphosphine (41 mg, 0.20 mmol) in dry dichloromethane (3 mL) at 0 °C, in nitrogen atmosphere. After 2 h, the reaction mixture was allowed to room temperature, finally it was concentrated under vacuum. No desired product was recovered but only degradation products of the starting materials.

*[(4-Methoxy-1-benzothiophen-2-yl)imino]-triphenylphosphorane* **22a**. This compound was prepared from the azide **20a** (100 mg, 0.49 mmol) and triphenylphosphine (128 mg, 0.49 mmol) following a reported procedure [38]. Chromatography on silica gel with petroleum ether/ethyl acetate 7:3 as eluent gave the title compound (72 mg, 33%). ^1^H-NMR (500 MHz, CDCl_3_) (ppm): δ_H_ 7.74–7.70 (m, 5H), 7.52–7.50 (m, 5H), 7.46–7.42 (m, 5H), 7.00–6.98 (d, 1H, *J_PH_* = 10 Hz), 6.88–6.85 (m, 1H), 6.51 (d, 1H, *J_PH_* = 5 Hz), 6.24 (s, 1H), 3.73 (s, 3H); ^13^C-NMR (125 MHz, CDCl_3_) (ppm): δ_C_ 161.2, 140.9, 132.6, 131.2, 128.7, 125.4, 123.3 121.4, 115.2, 108.6, 98.9, 56.2.

*[(4-[tert-Butyl(dimethyl)silyl]oxy-1-benzothiophen-2-yl)imino]-triphenylphosphorane* **22b**. This compound was prepared from the azide **20b** (66 mg, 0.22 mmol) and triphenylphosphine (59 mg, 0.22 mmol) following a reported procedure [38]. Chromatography on silica gel with petroleum ether/ethyl acetate 7:3 as eluent gave the title compound (24 mg, 20%) as a brown/green oil. ^1^H-NMR (500 MHz, CDCl_3_) (ppm): δ_H_ 7.74–7.70 (m, 5H), 7.50–7.49 (m, 5H), 7.42–7.41 (m, 5H), 7.01 (d, 1H, *J_PH_* = 11 Hz), 6.80–6.76 (m, 1H), 6.46 (d, 1H, *J_PH_* = 7 Hz), 6.03 (s, 1H), 0.95 (s, 9H), 0.1 (s, 6H); ^13^C-NMR (125 MHz, CDCl_3_) (ppm): δ_C_ 185.8, 166.9, 147.2, 135.5, 134.5, 132.9, 132.8, 132.7, 128.8, 128.7, 120.8, 114.7, 112.9, 80.3, 25.8, 23.8, 18.1, 1.0, −4.4.

*Tert-butyl (2((triphenylphosphoranylidene)amino)benzo[b]thiophen-4-yl)carbamate* **22c**. Compound **20c** (105 mg, 0.4 mmol) in dry dichloromethane (5 mL) was added dropwise to a solution of triphenylphosphine (117 mg, 0.44 mmol) in dry dichloromethane (5 mL) at 0 °C, in nitrogen atmosphere. After 2 h, the reaction mixture was allowed to reach room temperature and stirred over night; finally it was concentrated under vacuum. No desired product was recovered but only degradation products of the starting materials.

### 5.5. General Procedure for Synthesis of (1)Benzothieno[2,3-b]pyridines ***23a,b, 24a***

A solution of the appropriate iminophosphorane **22a,b** (0.12 mmol) in dry chloroform (3 mL) was treated with methyl *trans*-4-oxo-2-pentenoate (0.12 mmol) and then stirred in an inert atmosphere for 34 h at 45 °C. After removal of the solvent, the crude product was chromatographed on silica gel, using petroleum ether/ethyl acetate 7:3 as eluent. For yields see Table 1.

*Methyl 5-methoxy-2-methyl(1)benzothieno[2,3-b]pyridine-4-carboxylate* **23a–24a**. This compound was obtained as a thick oil. (Found: C, 62.72; H, 4.58; N, 4.85; S, 11.13%. C_15_H_13_NO_3_S requires C, 62.70; H, 4.56; N, 4.87; S, 11.16%); ^1^H-NMR (300 MHz, CDCl_3_) (ppm): δ_H_ 7.75–7.70 (m, 1H), 7.57–7.50 (m, 1H), 7.47–7.40 (m, 1H), 6.95–6.90 (m, 1H), 3.98 (s, 6H), 2.72 (s, 3H);^ 13^C-NMR (75 MHz, CDCl_3_) (ppm): δ_C_ 169.4, 167.8, 156.8, 139.4, 138.2, 132.5, 131.2, 129.1, 128.8, 118.2, 115.6, 106.6, 55.9, 30.3; MS: m/z = 287 (M^+^).

*Methyl 5-[tert-butyl(dimethyl)silyl]oxy-2-methyl(1)benzothieno[2,3-b]pyridine-4-carboxylate* **23b**. This compound was obtained as a thick oil. (Found: C, 62.00; H, 6.53; N, 3.58; S, 8.24%. C_20_H_25_NO_3_SSi requires: C, 61.98; H, 6.50; N, 3.61; S, 8.27%); ^1^H-NMR (300 MHz, CDCl_3_) (ppm): δ_H_ 7.50–7.25 (m, 3H), 7.00–6.95 (m, 1H), 3.90 (s, 3H), 2.72 (s, 3H), 0.81 (s, 9H), 0.15 (s, 6H); ^13^C-NMR (75 MHz, CDCl_3_) (ppm): δ_C_ 169.5, 154.2, 151.9, 150.5, 149.5, 141.5, 131.7, 128.4, 117.7, 117.0, 116.2, 53.9, 29.8, 26.2, 25.7, 19.8, 18.5, −0.4; MS: m/z: 387 (M^+^).

### 5.6. Synthesis of 4,6-Disubstituted Benzothieno[2,3-b]pyridine Precursors

*5-Methoxybenzo[b]thiophene* **25a**. Compound **25a** was prepared from commercial 4-methoxybenzenethiol (2 g, 14 mmol) according to a known procedure [57]. Chromatography on silica gel, using petroleum ether/ethyl acetate 9:1 as eluent, furnished the title compound **25a** as a thick fragrant yellow oil (218 mg, 31%). ^1^H-NMR (500 MHz, CDCl_3_) (ppm): δ_H_ 7.67 (d, 1H, *J_PH_* = 10 Hz), 7.37 (d, 1H, *J_PH_* = 5 Hz), 7.22–7.20 (m, 2H), 6.94–6.93 (d, 1H, *J_PH_* = 5 Hz), 3.81 (s, 3H); ^13^C-NMR (125 MHz, CDCl_3_) (ppm): δ_C_ 159.3, 141.0, 132.9, 127.9, 123.8, 123.6, 121.5, 106.7, 55.9; MS: m/z = 164 (M^+^).

*5-Hydroxybenzo[b]thiophene.* At 0 °C, to the substrate **25a** dissolved in chlorobenzene was added dropwise a solution of BBr_3_·S(CH_3_)_2_ 1 M in CH_2_Cl_2_. After about 30 min at 0 °C the solution is brought to reflux for 22 h. The reaction was quenched by adding about 50 mL of water and extracting the organic product three times with CH_2_Cl_2_ (25 mL). The combined organic phases were washed first with water, then with brine. The reaction product is purified by chromatography on silica gel (petroleum ether/diethyl ether 8:2 as eluent) to give 395 mg (81%) of 5-hydroxybenzo[*b*]thiophene as a light pink solid. Mp 89–92 °C. ^1^H-NMR (500 MHz, CDCl_3_) (ppm): δ_H_ 7.73 (d, 1H, *J_PH_* = 10 Hz ), 7.46 (d, 1H, *J_PH_* = 5 Hz), 7.27 (d, 1H, *J_PH_* = 5 Hz), 7.22 (d, 1H, *J_PH_* = 5 Hz); 6.94 (d, 1H, *J_PH_* = 5 Hz); 5.04 (s;1H). ^13^C-NMR (125 MHz, CDCl_3_) (ppm): δ_C_ 153.4, 141.1, 133.6, 128.1, 123.5, 116.2, 114.6, 108.8; MS: m/z 150 (M^+^).

*(Benzo[b]thiophen-5-yloxy)(tert-butyl)dimethylsilane* **25b**. 5-Hydroxybenzo[*b*]thiophene (320 mg, 2.13 mmol) was dissolved in 8 mL of dry dichloromethane and to this solution were added *tert*-butyldimethylsilylchloride (3.48 mmol), imidazole (6.68 mmol) and a catalytic amount of 4-dimethylaminopyridine. The resulting mixture was stirred in inert atmosphere for 3 h at room temperature, then was filtered and washed with saturated ammonium chloride solution and twice with water. After solvent removal the crude product was chromatographed on silica gel, using petroleum ether as eluent, to give **25b** as a thick light brown oil (434 mg, 83%). ^1^H-NMR (500 MHz, CDCl_3_) (ppm): δ_H_ 7.47 (d, 1H, *J_PH_* = 15 Hz), 7.19 (d, 1H, *J_PH_* = 15 Hz), 7.04 (d, 1H, *J_PH_* = 15 Hz), 7.02–6.99 (m, 1H), 6.70–6.68 (m, 1H), 0.79 (s, 9H), 0.30–0.00 (m, 6H); ^13^C-NMR (125 MHz, CDCl_3_) (ppm): δ_C_ 153.0, 140.9, 132.8, 127.3, 123.4, 122.9, 118.7, 113.5, 29.7, 25.7. MS: *m/z* 264 (M^+^); 207 (100).

*Tert-butyl benzo[b]thiophen-5-yl-carbamate* **25c**. To a solution of benzo[*b*]thiophen-5-amine (78 mg, 0.52 mmol) in dry dichloromethane (2 mL) was added di-*tert*-butyl dicarbonate (BOC) (148 mg, 0.68 mmol). The resulting mixture was stirred in an inert atmosphere for 15 h at room temperature. Then, after solvent removal, the crude was dissolved in ethyl acetate and the resulting organic phase was washed several times with water, once with brine and then dried over sodium sulphate. After solvent removal, the crude product was chromatographed on silica gel, using petroleum ether/ethyl acetate 7:3 as eluent, to give **25c** as a thick colourless oil (103 mg, 80%). ^1^H-NMR (400 MHz, CDCl_3_) (ppm): δ_H_ 8.01 (s, 1H), 7.76 (d, 1H, *J_PH_* = 8 Hz), 7.43 (d, 1H, *J_PH_* = 4 Hz); 7.26 (d, 1H, *J_PH_* = 4 Hz); 7.22 (d, 1H, *J_PH_* = 8 Hz), 6.64 (s, 1H), 1.56 (s, 9H); ^13^C-NMR (125 MHz, CDCl_3_) (ppm): δ_C_ 153.0, 140.3, 135.2, 134.4, 127.3, 123.8, 122.6, 116.8, 113.0, 80.5, 29.3. MS: *m/z* 249 (M^+^); 193 (100).

### 5.7. Synthesis of Azides ***26a, 26b, 26c***

*2-Azido-5-methoxy-1-benzothiophene* **26a**. Compound **25a** (300 mg, 1.8 mmol) in dry diethyl ether (4 mL) was treated, in an inert atmosphere, with 1.6M *n*-butyllithium in hexane (2.7 mmol) and refluxed for 1 h. Then the reaction mixture was cooled to −70 °C and tosyl azide (380 mg, 1.9 mmol) in dry diethyl ether (4 mL) was added dropwise. After 4 h at this temperature, the obtained triazene salt was filtered under vacuum, washed with dry diethyl ether and then treated at 0 °C with an aqueous solution of sodium pyrophosphate decahydrate (868 mg, 1.9 mmol, in 9 mL of water). After 15 min of stirring at this temperature the suspension was filtered on a Buckner filter and extracted twice with diethyl ether and then with ethyl acetate until the organic phase appeared colourless. Then, after solvent removal, the crude product was purified by chromatography on Florisil (eluent petroleum ether/ethyl acetate 8:2) giving the title compound (206 mg, 55%) as a thick orange oil. Azide **26a** is unstable; it was characterized by IR and ^1^H-NMR spectroscopy. ΙR ν_max_/cm^−1^ 2117 (N_3_); ^1^H-NMR (400 MHz, CDCl_3_) (ppm): δ_H_ 7.35–7.20 (m, 1H), 7.07 (s, 1H), 6.95 (d, 1H, *J_PH_* = 24 Hz), 6.75 (s, 1H), 3.84 (s, 3H).

*((2-Azidobenzo[b]thiophen-5-yl)oxy)(tert-butyl)dimethylsilane* **26b**. Compound **25b** (434 mg, 1.64 mmol) in dry diethyl ether (9 mL) was treated, in an inert atmosphere, with 1.6 M *n*-butyllithium in hexane (1.64 mmol) and refluxed for 1 h. Then the reaction mixture was cooled to −78 °C and, slowly, tosylazide (378 mg, 1.92 mmol) in dry diethyl ether (9 mL) was added. After 5 h at this temperature the obtained triazene salt was filtered on a Buckner filter, washed with dry diethyl ether and then treated at 0 °C with an aqueous solution of sodium pyrophosphate decahydrate (0.9 g, 2.02 mmol) in water (9 mL). After 15 min of stirring at this temperature the suspension was filtered on a Buckner filter and extracted twice with diethyl ether and then with ethyl acetate until the organic phase appeared colourless. Then, after solvent removal, the crude product was purified by chromatography on Florisil (eluent petroleum ether) giving the title compound (141 mg, 28%) as a thick yellow oil. Azide **26b** is unstable; it was characterized by IR and ^1^H-NMR spectroscopy. IR ν_max_/ cm^−1^ 2116 (N_3_). ^1^H-NMR (500 MHz, CDCl_3_) (ppm): δ_H_ 7.72–7.70 (d, 1H, *J_PH_* = 10 Hz), 7.43 (d, 1H, *J_PH_* = 10 Hz ), 7.27 (s, 1H), 6.73 (s, 1H), 1.03 (s, 9H), 0.23 (m, 6H).

*Tert-butyl (2-azidobenzo[b]thiophen-5-yl)carbamate* **26c**. Compound **24c** (141 mg, 0.56 mmol) in dry diethyl ether (3 mL) was treated, under a nitrogen atmosphere, with 1.6 M *n*-butyllithium in hexane (500 μL, 0.70 mmol) and refluxed for 1 h. Then the reaction mixture was cooled to −78 °C and tosylazide (135 mg, 0.68 mmol) in dry diethyl ether (3 mL) was added dropwise. After 5 h at this temperature, the reaction mixture was treated at 0 °C with an aqueous solution of sodium pyrophosphate decahydrate (412 mg; 0.92 mmol, in 3 mL of water). After 15 min of stirring at this temperature the suspension was extracted several times with diethyl ether and then with ethyl acetate until the organic phase appeared colourless and dried over sodium sulphate. Then, after solvent removal, the crude product was purified by chromatography on Florisil (eluant petroleum ether/diethyl ether 8:2) giving the title compound (150 mg, 62%) as a thick yellow oil. IR ν_max_/cm^−1^ 2126 (N_3_). ^1^H-NMR (400 MHz, CDCl_3_) (ppm): δ_H_ 8.01 (s, 1H), 7.86 (d, 1H, *J_PH_* = 8 Hz), 7.41 (s, 1H), 7.21 (d, 1H, *J_PH_* = 4 Hz), 6.55 (s, 1H), 1.55 (s, 9H). ^13^C-NMR (100 MHz, CDCl_3_) (ppm): δ_C_ 152.5, 140.3, 135.2, 134.2, 123.8, 122.7, 116.8, 113.0, 98.8, 80.6, 28.4.

### 5.8. Synthesis of Imminophosphoranes ***27a,b,c-28a,b***

*[(5-Methoxy-1-benzothiophen-2-yl)imino]-(methyl)diphenylphosphorane* **27a**. Compound **26a** (46 mg, 0.22 mmol) in dry diethyl ether (2 mL) was added dropwise to a solution of methyldiphenylphosphine (44 mg, 0.22 mmol) in dry diethyl ether (2 mL) at 0 °C, in nitrogen atmosphere. After 2 h, the reaction mixture was allowed to room temperature for another hour and then was concentrated under vacuum. The residual material was purified by chromatography on silica gel (eluent petroleum ether/ethyl acetate 7:3). The title compound (83 mg, 58%) was obtained as a brown solid. Mp 135–136°C. ^1^H-NMR (500 MHz, CDCl_3_) (ppm): δ_H_ 7.71–7.66 (m, 5H), 7.47–7.45 (m, 5H), 7.41–7.40 (m, 1H), 7.18 (d, 1H, *J_PH_* = 15 Hz), 6.72 (s, 1H), 6.52 (d, 1H, *J_PH_* = 10 Hz), 5.88 (s, 1H), 3.70 (s, 3H), 2.08 (d, 3H, *J_PH_* = 15 Hz); ^13^C-NMR (100 MHz, CDCl_3_) (ppm): δ_C_ 159.6, 157.5, 142.6, 132.5, 131.8, 131.7, 130.8, 129.8, 129.3, 129.1 126.4, 122.1, 109.4, 105.3, 105.2, 105.1, 103.6, 55.7, 15.1.

*5-((Tert-butyldimethylsilyl)oxy)-N-(methyldiphenylphosphoranylidene)benzo[b]thiophen-2-amine* **27b**. Compound **26b** (62 mg, 0.21 mmol) in dry dichloromethane (2 mL) was added dropwise to a solution of methyldiphenylphosphine (42 mg, 0.21 mmol) in dry dichloromethane (2 mL) at 0 °C, in nitrogen atmosphere. After 2 h, the reaction mixture was taken to room temperature for another hour and then was concentrated under vacuum. The residual material was crystallized from diethyl ether and characterized. The title compound (79 mg, 79%) was obtained as a green thick oil. ^1^H NMR (500 MHz, CDCl_3_) (ppm): δ_H_ 7.58–7.56 (m, 4H), 7.39–7.37 (m, 4H), 7.21–7.19 (m, 2H), 6.82 (d, 1H, *J_PH_* = 5 Hz), 6.73 (s, 1H), 6.44 (d, 1H, *J_PH_* = 10 Hz), 5.90 (s, 1H), 2.23 (d, 3H, *J_PH_* = 10 Hz), 0.83 (s, 9H), 0.19 (d, 6H); ^13^C NMR (125 MHz, CDCl_3_) (ppm): δ_C_ 155.0, 140.8, 132.8, 131.3, 130.1, 128.70, 127.5, 123.3, 114.6, 98.9, 30.2, 25.9, 25.3.

*Tert-butyl(2-((methyldiphenylphosphoranylidene)amino)benzo[b]thiophen-5-yl)carbamate* **27c**. Compound **26c** (100 mg, 0.34 mmol) in dry dichloromethane (3 mL) was added dropwise to a solution of methyldiphenylphosphine (85 mg, 0.42 mmol) in dry dichloromethane (3 mL) at 0 °C, in nitrogen atmosphere. After 2 h, the reaction mixture was taken to room temperature for another hour and then was concentrated under vacuum. The residual material was crystallized from diethyl ether and characterized. The title compound (77 mg, 49%) was obtained as white needles. Mp 112–114 °C. ^1^H-NMR (500 MHz, CDCl_3_) (ppm): δ_H_ 8.58 (s, 1H), 7.81 (d, 1H, *J_PH_* = 10 Hz), 7.73–7.62 (m, 5H) 7.54–7.39 (m, 5H), 7.23 (s, 1H), 6.98–6.97 (d, 1H, *J_PH_* = 5 Hz), 5.18 (s, 1H), 2.38 (s, 3H), 2.27 (s, 9H). ^13^C-NMR (125 MHz, CDCl_3_) (ppm): δ_C_ 152.5, 143.0, 140.0, 137.0, 135.5, 129.2, 128.2, 126.3, 123.3, 123.0, 116.4, 115.9, 98.8, 79.5, 38.7, 28.4, 25.2.

*[(5-Methoxy-1-benzothiophen-2-yl)imino]-triphenylphosphorane* **28a**. Compound **26a** (125 mg, 0.61 mmol) in dry diethyl ether (5 mL) was added dropwise to a solution of triphenylphosphine (162 mg, 0.62 mmol) in dry diethyl ether (5 mL) at 0 °C, in nitrogen atmosphere. After 2 h, the reaction mixture was allowed to room temperature, then was concentrated under vacuum. The residual material (a very dark and thick oil) was purified by chromatography on silica gel (eluent petroleum ether/ethyl acetate 8:2). The title compound (200 mg, 74%) was obtained as a brown oil. ^1^H-NMR (500 MHz, CDCl_3_) (ppm): δ_H_ 7.81–7.77 (m, 5H), 7.61–7.57 (m, 5H), 7.52–7.47 (m, 5H), 7.3 (s, 1H), 6.80 (d, 1H, *J_PH_* = 5 Hz), 6.63–6.60 (m, 1H), 6.07 (s, 1H), 3.78 (s, 3H); ^13^C-NMR (100 MHz, CDCl_3_) (ppm): δ_C_ 162.2, 160.5, 144.2, 136.3, 134.8, 132.7, 131.0, 129.7, 129.5, 129.2, 126.8, 122.7, 109.1, 105.7, 105.4, 105.1, 103.1.

*5-((Tert-butyldimethylsilyl)oxy)-N-(triphenylphosphoranylidene)benzo[b]thiophen-2-amine* **28b**. Compound **26b** (66 mg, 0.22 mmol) in dry dichloromethane (2 mL) was added dropwise to a solution of triphenylphosphine (60 mg, 0.23 mmol) in dry dichloromethane (2 mL) at 0 °C, in nitrogen atmosphere. After 2 h, the reaction mixture was taken to room temperature for another hour and then was concentrated under vacuum. The crude was purified on silica gel using petroleum ether/dichloromethane 1:1 as eluent to give the title compound (97 mg, 81%) as a yellow solid. Mp 163–165 °C. ^1^H-NMR (500 MHz, CDCl_3_) (ppm): δ_H_ 7.82–7.78 (m, 5H), 7.60–7.50 (m, 10H), 7.23 (d, 1H, *J_PH_* = 10 Hz), 7.01 (s, 1H), 6.75 (s, 1H), 6.53 (d, 1H, *J_PH_* = 10 Hz), 0.99 (s, 9H), 0.19 (m, 6H); ^13^C-NMR (125 MHz, CDCl_3_) (ppm): δ_C_ 152.6, 132.9, 132.9, 132.6, 130.8, 128.8, 125.3, 121.6, 114.5, 112.5, 98.8, 29.7, 25.8.

### 5.9. General Procedure for the Synthesis of (1)-Benzothieno[2,3-b]pyridines ***29a,b, 30a,b***

A solution of the appropriate iminophosphorane **27a,b, 28a,b** in dry chloroform was treated with methyl *trans* 4-oxo-2-pentenoate (1.1 eq) and then stirred in inert atmosphere at 61 °C. After removal of the solvent, the crude product was chromatographed on silica gel, using petroleum ether/ethyl acetate 9:1 as eluent. For yields see Table 2.

*Methyl 6-methoxy-2-methyl(1)benzothieno[2,3-b]pyridine-4-carboxylate* **29a-30a**. This compound was obtained as a thick oil. ^1^H-NMR (500 MHz, CDCl_3_) (ppm): δ_H_ 7.95 (s, 1H), 7.75 (d, 1H, *J_PH_* = 12 Hz), 7.47 (s, 1H), 7.09–7.07 (d, 1H, *J_PH_* = 12 Hz), 4.10 (s, 3H), 3.91 (s, 3H), 2.74 (s, 3H); ^13^C-NMR (125 MHz, CDCl_3_) (ppm): δ_C_ 168.2, 160.0, 160.3, 142.4, 136.6, 133.5, 132.2, 131.2, 129.5, 126.4, 120.9, 115.6, 106.5, 55.8, 27.5; MS: *m/z* 287 (M^+^).

*Methyl 6-((Tert-butyldimethylsilyl)oxy)-2-methylbenzo[4,5]thieno[2,3-b]pyridine-4-carboxylate* (**29b-30b**). Thick red oil. ^1^H-NMR (500 MHz, CDCl_3_) (ppm): δ_H_ 7.84 (s, 1H); 7.72–7.71 (m, 1H), 7.43 (s, 1H), 7.07 (d, 1H, *J_PH_* = 5 Hz), 4.09 (s, 3H), 2.74 (s, 3H), 1.05 (s, 9H), 0.35 (s, 6H); ^13^C-NMR (125 MHz, CDCl_3_) (ppm): δ_C_ 165.9, 159.0, 151.1, 142.8, 133.6, 132.9, 127.5, 126.5, 124.9, 120.9, 114.6, 112.6, 51.6, 30.2, 25.9, 23.5, −0.9; MS: *m/z* 387 (M^+^).

## 6. Conclusion

In summary, in this paper we have examined the general characteristics of peptidomimetics and pseudopeptides: Their use as drugs is under development. Particular attention has been devoted to the synthesis of tricyclic structures as potential scaffolds for new pseudopeptides. Moreover, the choice of 4- and 5-substituted benzothiophenes is relevant, not only for their isosterism with biologically active compounds containing indole rings, but also for the electronic characteristics of sulfur in interaction with the active sites of biological molecules.
